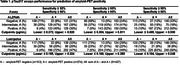# Approach for developing interpretative cut‐points during implementation of a plasma p‐tau217 assay in the clinical laboratory

**DOI:** 10.1002/alz.094609

**Published:** 2025-01-09

**Authors:** Alicia Algeciras‐Schimnich

**Affiliations:** ^1^ Mayo Clinic, Rochester, MN USA

## Abstract

**Background:**

Implementation of Alzheimer disease blood‐based biomarkers (AD BBBs) in the clinical laboratory requires careful evaluation of test performance and selection of interpretative cut‐points to prevent patient misclassification that may lead to further evaluation, misdiagnosis, and/or potentially unnecessary treatment.

**Method:**

Participants with mild cognitive impairment or mild dementia (n = 427) and with an EDTA‐plasma sample available within 6 months of amyloid‐PET imaging were selected through the Mayo Clinic Study of Aging and the Mayo Clinic Alzheimer’s Disease Research Center in Rochester, Minnesota. Amyloid‐PET was performed with ^11^C‐Pittsburg Compound B; abnormal amyloid (A+) was defined as Centiloid ≥25 (SUVR≥1.52) (n = 274 A+, 153 A−). EDTA‐plasma samples were measured using the ALZpath Simoa pTau217 assay and the Fujirebio Lumipulse pTau217, Aβ42/Aβ40 and pTau217/Aβ42 ratio assays. The clinical performance of a 1‐cutpoint as well as a 2‐cutpoint model that incorporates an intermediate range were assessed. For the 2‐cutpoint model, various combinations of sensitivity and specificity were examined to identify thresholds that maximize overall test performance.

**Result:**

While both pTau217 assays displayed high ROC AUC (>0.90), neither assay achieved ≥90% in both sensitivity and specificity using a 1‐cutpoint model. Using a 2‐cutpoint model for the Lumipulse pTau217 assay, the combination of 92% sensitivity and 96% specificity to define lower and upper cutpoints provided the optimal balance of false positives and false negatives, while keeping 20% or fewer results categorized as intermediate. For the ALZpath assay, 92% sensitivity and 96% specificity could only be achieved with 39% of the patients in this cohort falling into the intermediate range. Using this approach, the Lumipulse pTau217 assay would reduce more costly CSF or PET testing by 80%.

**Conclusion:**

Cutpoints for the identification of amyloid pathology were defined in a cohort of individuals with MCI and mild dementia. Information obtained on the performance of these defined cut‐points was used to select the most appropriate assay and cut‐points to implement in clinical practice.